# Trace Metal Lead Exposure in Typical Lip Cosmetics From Electronic Commercial Platform: Investigation, Health Risk Assessment and Blood Lead Level Analysis

**DOI:** 10.3389/fpubh.2021.766984

**Published:** 2021-11-17

**Authors:** Yanan Li, Yanyan Fang, Zehua Liu, Yahan Zhang, Kangli Liu, Luping Jiang, Boyuan Yang, Yongdie Yang, Yongwei Song, Chaoyang Liu

**Affiliations:** ^1^Research Center for Environment and Health, Zhongnan University of Economics and Law, Wuhan, China; ^2^School of Business Administration, Zhongnan University of Economics and Law, Wuhan, China; ^3^Department of Environmental Science and Engineering, Zhongnan University of Economics and Law, Wuhan, China; ^4^Renmin Hospital of Wuhan University, Wuhan, China

**Keywords:** lip cosmetics, Pb, health risk, Python crawler, Monte Carlo, Chinese e-commerce market

## Abstract

Lead (Pb) in lipstick products has become an increasing concern, which can cause safety problems to human body directly with diet. To investigate the Pb exposure and potential health risk level of typical popular lip cosmetics in Chinese e-commerce market, Python crawler was introduced to identify and select 34 typical popular lip cosmetics, including 12 lipsticks, 13 lip glosses, and 9 lip balms. And then this study used ICP-MS to determine the content of Pb. Furthermore, the ingestion health risk assessment method issued by United States Environmental Protection Agency (USEPA) and Monte Carlo simulation algorithm were applied to assess the probabilistic health risks of adults exposure. Finally, taking the possible exposure of children contacting with lip products, the health risk assessment of children blood Pb was carried out. The results showed that the concentration of Pb in lip products ranged from 0 to 0.5237 mg/kg, which was far lower than the limit set by various countries. The probabilistic non-carcinogenic risks and carcinogenic risks were 4.93 ×10^−7^~2.82 ×10^−3^ and 1.68 ×10^−12^~9.59 ×10^−9^, respectively, which were in an acceptable level. The results of blood Pb assessment suggested that the Pb content of lip cosmetics had no obvious influence on blood Pb concentration of children, and background Pb exposure is the main factor affecting children's blood Pb level (BLL). Overall, the samples of lip products are selected by Python crawler in this study, which are more objective and representative. This study focuses on deeper study of Pb, especially for the health risk assessment of blood Pb in children exposed to lip products. These results perhaps could provide useful information for the safety cosmetics usage for people in China and even the global world.

## Introduction

In recent years, the security of cosmetics is increasingly recognized as a worldwide public health concern (1–3). The Food and Drug Administration (FDA) of the United States, the Food and Drug Safety Administration of the ROK, and the National Drug Administration of China have successively reported that the problem of excessive heavy metals in cosmetics is serious (4–7). To improve product performance, heavy metals are added to cosmetics by manufacturers. For example, the addition of Pb, Hg (mercury), and other metals makes cosmetics have the whitening function of covering or inhibiting pigment formation (8). In addition, with the aggravation of environmental pollution and the influence of some natural factors, some heavy metals widely existing in the surrounding environment will also enter people's lives with the use of cosmetic raw materials (9–11). Studies have shown that low-dose but long-term exposure of heavy metals to the human body can cause chronic poisoning or even canceration, causing irreversible adverse effects on human organs and seriously threatening human health (12–14). As one of the most commonly used cosmetics for modern women, lip cosmetics can not only moisten the lips but also improve personal performance (15). Compared with other cosmetics, lip products are easily exposed to the human body directly with the diet, and result in a higher health risk. Therefore, the assessment of exposure levels and health risks of heavy metals in lip products would be of great value to design manufacturers and relevant supervisory departments to provide scientific reference for health risk control of lip cosmetics consumers.

At present, some studies have focused on heavy metal pollution and health risks in lip products (16–18). Al-Saleh et al. measured the contents of various heavy metals in lipsticks obtained from offline markets in different years, the results indicated that the content of Pb was lower than the standard set by FDA, but special attention should be paid to the effect of Ni (nickel) and Cr (chromium) on allergic dermatitis (19, 20). Pinto et al. investigated the contents of 44 elements in 96 lipsticks from Brazil and Portugal, the results ranged from <1 μg/g to a few tens of μg/g (such as Zn, Mn, Pb) (21). Gao et al. simulated the absorption of eight heavy metals in lip cosmetics by hydrochloric acid dilution method, vitro gastrointestinal method and American Pharmacopeia method, and the results suggested that Cu (copper), Pb and Cd (cadmium) were the main components of heavy metal pollution (22). Kilic et al. analyzed the lipsticks collected from the local markets in Turkey, and found the concentrations of all heavy metal were below the allowable limits (18). Although these researches are widely investigated the contents of various heavy metal, few studies focus on the deep study of a specific heavy metal element. Hence, this study attempted to select the element of Pb for deeper study. As a kind of 2B carcinogen, once Pb is absorbed by human body for about 3 months, the concentration of blood Pb will reach balance. Then, the majority of Pb is stored in the liver and the littleness left in the kidney, while the rest Pb is dispersed throughout the body (spinal cord, adrenal gland, brain, heart, etc.) (23). Excessive Pb in the human body can initiate gastrointestinal diseases, even poison the liver, central nervous system, and cause other serious harm to human health (24–27). The experts of WHO have stressed the significance of control on Pb in children, since the disadvantageous effects of Pb on central nervous system (28). Particularly for children under 6 years old, Pb is more harmful because it can damage memory, reduce intelligence, hinder growth and development (29). As mentioned above, Pb is significant among many heavy metals (30, 31), and it is high time to investigate the exposure of Pb to human body in lip products. What's more, some studies have shown that the contents of Pb and Cr in lip cosmetics exceed the standard, and it is strongly recommended to pay concern to the health risks of lip cosmetics (18, 22, 32). The contents of Pb in lip cosmetics from local supermarkets in Malaysia were 0.77–15.44 mg/kg (33). In South Korean, the maximum contents of Pb in lip products from local supermarkets were 12.77 mg/kg (34). A result, from a survey of lipstick from Portuguese and Brazilian markets, showed that the systemic exposure of lipstick was <0.2% of the daily allowable exposure, except for Pb (21). Alnuwaiser detected the content of Pb in the lipstick of Chinese products, there were only three samples above the limit, while the Pb level in other groups between 0.7 and 12.34 ppm (35). Feizi et al. found the concentrations of Pb were higher than those of Cd in lipsticks, only 33% of lipsticks had Pb contents less than the FDA limit (36). In Turkey, Kilic et al. found the concentration of Pb in Lipstick was 1.1 mg/kg (18). Obviously, the contents of Pb and corresponding health risks have been shown to vary in many different lip products and countries, and there have been little deep discussion about the health risk analysis. Therefore, this study attempts to focus on deeper study of the health risk assessment of Pb of typical popular lip cosmetics in Chinese e-commerce market.

Besides, the assessment results are uncertain due to the differences in sample distribution and exposure parameters during the actual risk assessment process (13, 37–39). To deal with the uncertainty of sample distribution, this study introduced Monte Carlo simulation to analyze the distribution of sample contents. As a statistical simulation method, the Monte Carlo simulation can descent the uncertainty of the mean value under complex condition, and get more accurate prediction results (40–42). The essence of Monte Carlo simulation is to simulate the possible random phenomena in the actual system by random numbers which obey a certain distribution. And the basic idea is to input abundant of random numbers satisfying a certain probability distribution into the model as parameters to determine the probability distribution of the concerned variables (43, 44). Combined with the Monte Carlo simulation and the health risk assessment model recommended by USEPA, the corresponding probability risk assessment range is further obtained.

Of particular concern is Pb mainly existing in the form of blood Pb in the human body (45). Kinds of pollution factors in the environment may result in the increase of blood Pb concentration, such as the Pb pollution in soil (46, 47). In recent years, some studies have begun to pay attention to the blood Pb problem of children caused by the use of lip cosmetics (48). Among them, there are mainly two pathways for children to expose. One is that children directly use lip balm, and the other is that Pb in lip products will also be released into children through maternal transmission (49). At present, several methods currently exist for the measurement of health risks of blood Pb, mainly include Adult Lead Model (ALM) and Integrated Exposure Uptake Biokinetic Model (IEUBK) (48, 50, 51). Among them, the IEUBK mainly involves children aged 0~7. These two models not only consider the existence and quantity of corresponding harmful substances in the health risk assessment of blood Pb, but also combine the estimated background exposure and the comparison with the basic health standards (52). Through the health risk assessment of adults and blood Pb in children exposed to lip products, this study perhaps could provide useful information for the safety cosmetics usage for people in China and even the global world.

In brief, this study selected 34 typical best-selling lip cosmetics from Chinese e-commerce market based on Python crawler technology, and analyzed the health risk level of adults and children caused by Pb exposure of lip products. The main objectives were (i) to investigate the popular lip cosmetics in Chinese e-commerce market, and determine the content of heavy metal Pb; (ii) to estimate the non-carcinogenic and carcinogenic risks of lipstick users with different frequencies using the health risk assessment model recommended by USEPA; (iii) to evaluate the effects of lip cosmetics on children's blood Pb content by ALM and IEUBK.

## Materials and Methods

### Samples Selection

**Figure 1:**
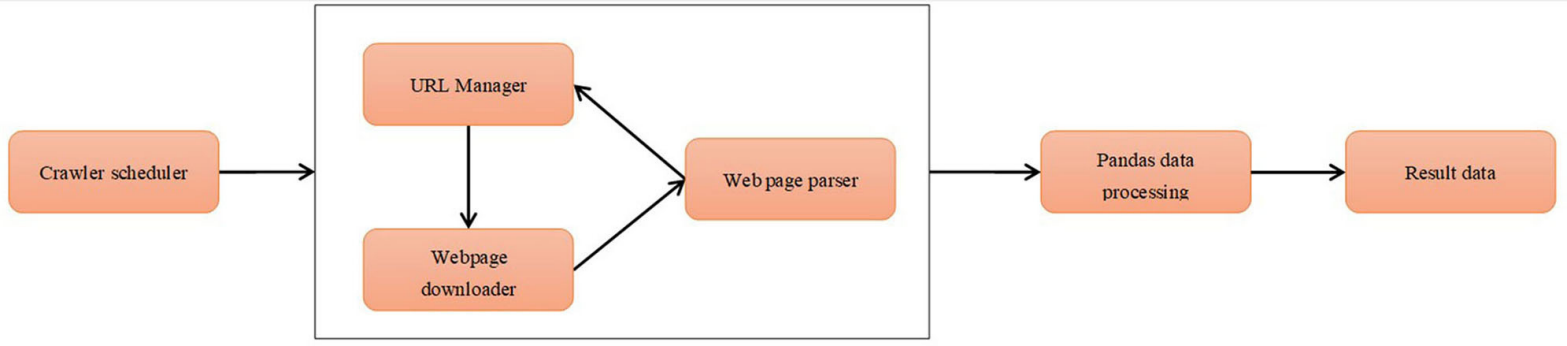
The crawling process by Python.

With the generality of online shopping in Chinese market, this study used Python crawler technology to select the most popular lip products in Chinese typical e-commerce market. JingDong, as one of Chinese top ten e-commerce markets, was selected as the research object of this study. The main Python crawler steps were shown in Figure 1. The first step was to establish the initial URL link: www.jingdong.com. Links unrelated to the topic were filtered based on the webpage analysis algorithm, while the useful links were retained and put into the URL pool. The second step was to start the crawler scheduler and initialize the Python crawler. Then, the crawler program simulated the browser to send a request “response” to obtain the HTML file of the corresponding link in the URL pool, and the server sent the “response” file object back to the browser. Python crawler called the BeautifulSoup library to parse HTML in “response.” Next, it parsed the Web page according to HTML syntax and accessed valuable data. The third step, Pandas was used to analyze and obtain the value data after the end of the crawler data process, and got the analysis results finally.

**Figure 2:**
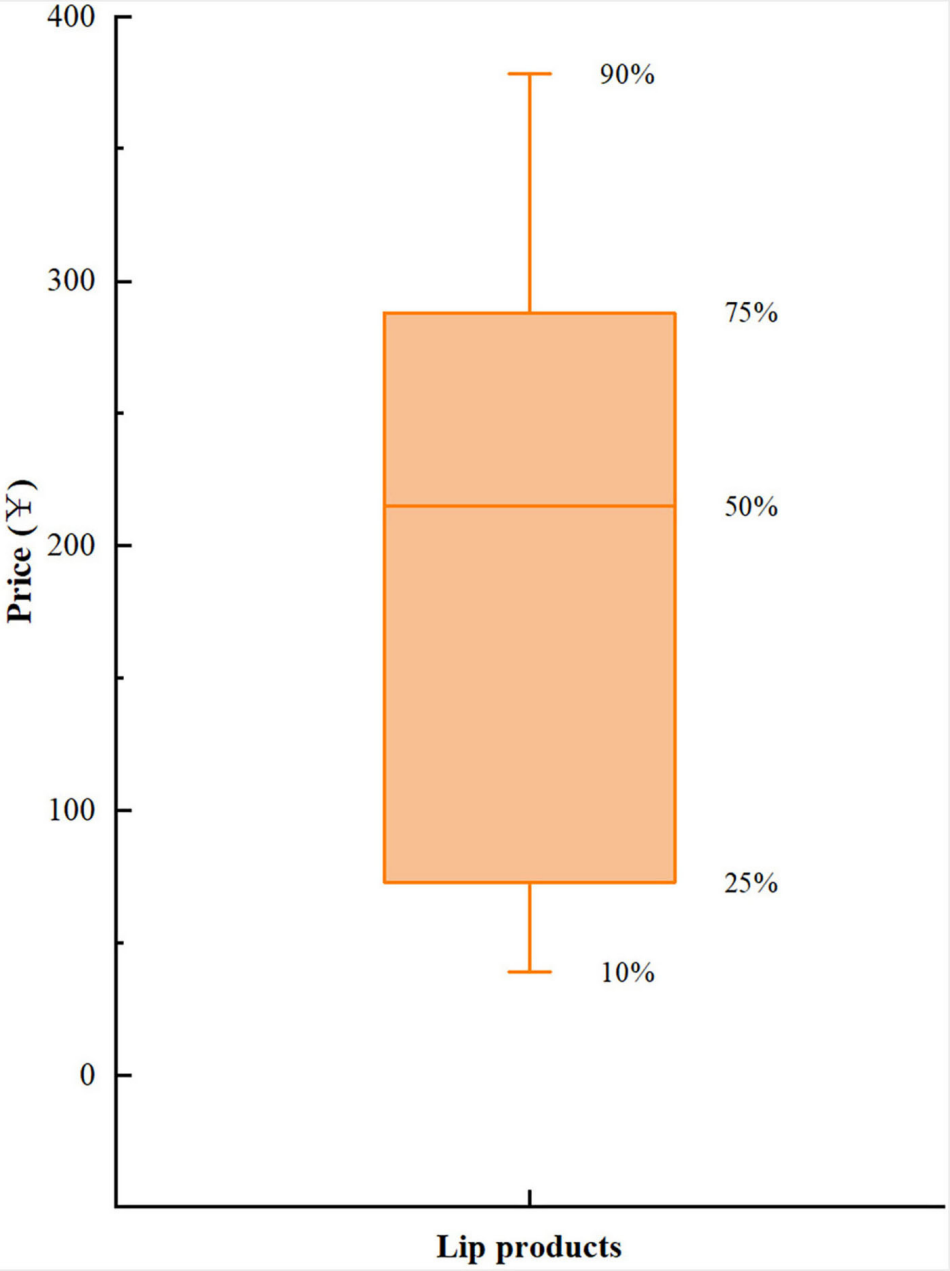
The boxplot of price distribution.

As shown in Figure 2, the box price distribution of lip cosmetics was obtained on the basis of crawler data. The price distribution of lip cosmetics mainly ranged from 0 to 400 RMB, so the study classified the unified brand of lip cosmetics according to the 33th percentiles and the 66th percentiles, 0~95 RMB, 96~266 RMB, and more than 267 RMB, respectively.

### Samples Collection and Analysis

#### Samples Collection

Based on the crawler analysis results above, the top lipstick brands were selected from each section by combining market share and commodity reviews. A total of 34 samples were purchased in this study, including lipsticks (LS, *n* = 12), lip glosses (LG, *n* = 13) and lip balms (LB, *n* = 9). Detailed information involves sample number, brand, origin, color, and price are shown in Table 1.

**Table 1:** The basic information for all samples (*N* = 34).

**Category**	**Number**	**Brand**	**Production Country**	**Color**	**Price (RMB)**
Lipsticks	LS1	Chanel	France	Orange red	320
(*n* = 12)	LS2	YSL	France	True red	320
	LS3	Armani	France	True red	320
	LS4	Dior	France	True red	330
	LS5	Dhc	Japan	Orange	158
	LS6	Mac	Canada	Brick red	185
	LS7	L′ Oreal	China	Orange red	135
	LS8	Maybelline	China	Orange red	120
	LS9	Carslan	China	Cameo brown	139
	LS10	Revlon	America	Orange red	78
	LS11	Kiko	Italy	Cameo brown	90
	LS12	Zeesea	China	Brick red	70
Lip glosses	LG1	YSL	France	Cameo brown	320
(*n* = 13)	LG2	Armani	France	Orange red	310
	LG3	Chanel	France	Purplish red	330
	LG4	Dior	France	True red	330
	LG5	Mac	Canada	Pink	170
	LG6	Maybelline	China	True red	122
	LG7	L′ Oreal	China	Cameo brown	145
	LG8	Carslan	China	Orange red	109
	LG9	Revlon	America	Cameo brown	48
	LG10	Chioture	China	Cameo brown	60
	LG11	Zeesea	China	Brick red	80
	LG12	Kiko	Italy	Pink	90
	LG13	Kiko	Italy	Colorless	90
Lip balms	LB1	Dhc	Japan	Colorless	78
(*n* = 9)	LB2	Uriage	France	Colorless	78
	LB3	Maybelline	China	Light red	30
	LB4	Mentholatum	China	Colorless	35
	LB5	Vaseline	America	Colorless	25
	LB6	Vaseline	America	Light red	25
	LB7	Herbacin	Germany	Colorless	45
	LB8	Mentholatum	China	Light red	36
	LB9	Burt's bees	America	Colorless	70

#### Sample Determination

The sample pretreatment experiment was carried out by wet digestion method according to *Safety specification for cosmetics* (2015 edition) (53). Firstly, 0.5000~1.0000 g of samples were weighed using EL204 electronic balance (Mettler-Toledo Co., Ltd.) and placed in the crucible. The samples were heated at 100°C using a DK-98-11A electrothermal constant temperature water bath (Tianjin Tester Instrument Co., Ltd.) to make all the samples flow into the bottom of the crucible. 10~15 ml mixed acid were added, which was mixed with high grade pure nitric acid (Kaifeng Dongda Chemical Co., Ltd.) and high grade pure perchloric acid (Tianjin Zhengcheng Chemical Products Co., Ltd.) at a ratio of 3:1. Heating digestion was carried out on DB-4A stainless steel electric heating plate (Changzhou Boyuan Experimental Analysis Instrument Factory) to produce white smoke in the digestion solution, and the crucible was shaken slowly from time to time to make the digestion solution uniform. When the acid solution is pale yellow or colorless, turn off the power supply of the electric heating plate. The liquid was diluted with ultra-pure deionized water to a final volume of 25 ml when the remaining solution was only about 2~3 ml. In this study, Pb in lip cosmetics was selected for determination. The pretreated samples, parallel samples, and blank samples were put into the preheating inductively coupled plasma mass spectrometer (Nexlon350x, PE, USA) to determine the concentrations of heavy metals. The instrument was calibrated with 187Re in the determination of heavy metals by ICP-MS.

In order to remove the background values of heavy metals in the reagents and containers, the samples were not exposed to metal containers during the experiment, and the required experimental containers were soaked in nitric acid for more than 24 h before drying and using. Besides, parallel samples were more than 20% level and set blank control throughout the experiment. In the determination experiment, Pb standard solution and blank solution were selected to control the metal concentration of the sample. The whole blank test results are less than the detection limit of the method to ensure that the sample is not contaminated during digestion and determination. The standard curve was drawn for each sample test, and the correlation coefficient of the standard curve was ≥0.995, and the relative deviation of the parallel sample test results was controlled within 10%.

### Health Risk Assessment of Pb Exposure

#### Carcinogenic and Non-carcinogenic Health Risk Assessment Models

Considering the particularity of lip products exposed to the human body through diet, this study selected the health risk assessment model recommended by USEPA for oral ingestion (54, 55). The calculation formula was shown in Equations (1-3).


(1) $$\begin{array}{rcl} ADDing & = & C\times IR\times EF\times ED\times CF/BW/AT \end{array}$$

(2) $$\begin{array}{rcl} HQ & = & ADD/RfD \end{array}$$

(3) $$\begin{array}{rcl} LCR & = & ADD\times SF \end{array}$$

Where ADD represents the average daily dose of Pb in lip products, mg/(kg·d). C is the concentration of Pb in lip products, mg/kg. All parameters are shown in Table 2, IR represents the intake rate of lip products, g/d. The Intake rate of average users and high users was 0.02578 and 0.14902 g/d, respectively (54). EF is the exposure frequency, set as 365 d/a (55). ED represents the exposure duration, 70a (55). CF represents the conversion rate, set to 0.001 (56). AT is the average exposure time, 25,550 d (56). BW is the average body weight of the exposed population, which is 60 kg (57). RfD is the non-carcinogenic reference dose, mg/(kg·d). The general toxicity data of IRIS of USEPA, shown in plenty of researches, can be applied not only for food (58–60), but also for soil (61, 62), dust (63, 64) or other media, such as lip products (22, 33, 65). Therefore, according to the previous studies (22, 66), it is appropriate to cite the general toxicity data of IRIS of USEPA as the reference dose (RfD) of metals in lip products. The RfD was set to 0.0004 in this study (67). SF is carcinogenic slope factor, (kg·d)/mg, set to 0.0085 (67). HQ and LCR are the non-carcinogenic risk value and carcinogenic risk value of oral intake of Pb, respectively. The reference values of non-carcinogenic risk and carcinogenic risk are 1 and 10^−6^, respectively. If the calculated results are greater than the corresponding value, it is considered that the risk is large, and certain control measures are needed to reduce the risk. Otherwise, it is considered that the risk is at an acceptable level (56, 57, 67).

**Table 2:** Parameter selection and references of health risk assessment model for oral ingestion.

**Parameter type**	**Parameter value**	**References**
Intake rate of average users IR(g/d)	0.02578	(54)
Intake rate of high users IR(g/d)	0.14902	(54)
Exposed frequency EF(d/a)	365	(55)
Exposure duration ED(a)	70	(55)
Turnover rate CF	0.001	(56)
Average exposure time AT(d)	25550	(56)
Body weight BW(kg)	60	(57)
Non-carcinogenic reference dose RfD (mg/(kg·d))	0.0004	(67)
Cancer slope factors SF ((kg·d)/mg)	0.0085	(67)

#### Blood Lead Level Model

ALM is mainly composed of three parts: namely soil exposure, lip cosmetics exposure, and reference value. The detailed expression is shown in formula (4).


$$\begin{array}{rcl} PbB & = & \left(Pbs\times BKSF\times IR_{\text{S}+\text{D}}\times AF_{\text{S},\text{D}}\times EFs/AT_{\text{S},\text{D}}\right) \end{array}$$

(4) $$\begin{array}{r} +\left(Pb_{\text{L}}\times BKSF\times IR_{\text{L}}\times AF_{\text{L}}\times EF_{\text{L}}/AT_{\text{L}}\right)+PbB_{0} \end{array}$$

In the formula, PbB represents adult blood Pb concentration, μg/dl. Pbs and Pb_L_ are BLL in soil and lip cosmetics, respectively, μg/g. According to the research results of scholars on the soil Pb reference value in China, the value of Pbs was set as 282 μg/g (68). Based on the experimental data in this study, the average concentration of Pb in three kinds of lip cosmetics was set to 0.05791 μg/g. PbB_0_ was taken 1.62μg/dl as the reference blood Pb content. BKSF is the biokinetic slope factor, set as 0.4 d/dl. IR represents daily intake, and soil intake IR_S+D_ is 0.05 g/d. For lip cosmetics intake, average users was 0.0258 g/d and high users was 0.1490 g/d (67). AF represents the absorption rate, AF_S, D_ for soil is 0.12. AF_L_ for lip cosmetics is conservatively considered to be 1. EF represents the contact frequency. EFs for soil is set to 220 d/year, and EF_L_ for lip cosmetics is set to 365 d/year (69). AT represents the average contact time, which is 365 d/year (69). When blood Pb in adults is transmitted through the mother, the transmission rate is 0.85 in ALM and 0.9 in IEUBK (48).

The IEUBK model for children exposed to Pb was developed by USEPA to predict the blood Pb concentration of children under 7 years old (0~84 months) and the possibility of Pb poisoning in children after a certain degree of Pb exposure (70–72). This model can set up integral and external parameters with the consideration of soil, water, food, air and conditions of Pb exposure. The integral parameters can't be changed arbitrarily, while the external parameters can be modified based on local actual data at the input interface to simulate the relationship between Pb absorption efficiency and blood Pb concentration in the human body (68, 73). Taking the actual situation of children's use of lip cosmetics into account, this study defaulted that children were exposed only to lip balms, and Pb exposure in other types of lip cosmetics was transmitted by the mother (48). The reference value of Pb concentration in soil in China is set at 282 mg/kg, and the default absorption rate of Pb in soil and dust by children is 45% (68, 69). In accordance with the national revised limit of Pb concentration in the atmosphere in 2012, the background value of Pb concentration in the air was set as an annual average of 0.5 μg/m^3^ (GB3095-2012) (74), and the default absorption rate was 30%. The background value of Pb in drinking water was 0.01 μg/L (GB5749-2006) (75), and the default values of 0~7 years old were 0.2, 0.5, 0.52, 0.53, 0.55, 0.58, and 0.59 L/day (69). The blood Pb concentration in pregnant women was set based on the results of the ALM model. In accordance with China standard on the limit of pollutant content in food (GB2762–2017) (76), the limit of Pb content in infant formula foods was 0.15 and 0.02 mg/kg with solid and liquid food, respectively. The limit of Pb content in infant auxiliary food was 0.2~0.3 mg/kg, and the limit of Pb content in food was below 0.5 mg/kg except for seafood. According to the pyramid distribution of children's food intake, the background value of Pb intake in children aged 0~3 was set to 8 μg/day, and 20 μg/day in children aged 3~7 (68, 69). The statistical software is IEUBK win1.1_Build 11 (77).

### Statistical Analysis

Microsoft Excel 2019 was used for data processing. The correlation analysis was performed in SPSS (20.0), and the significance level was *P* < 0.05. In order to quantify the uncertainty of sample content distribution, Crystal Ball software (16.0) was used for Monte Carlo simulation in this study, and the number of iterations was set to be 10,000. The box diagrams of probability risk were drawn in Origin Pro 8.0.

## Results and Discussion

### Pb Content Characteristics in Lip Cosmetics

**Figure 3:**
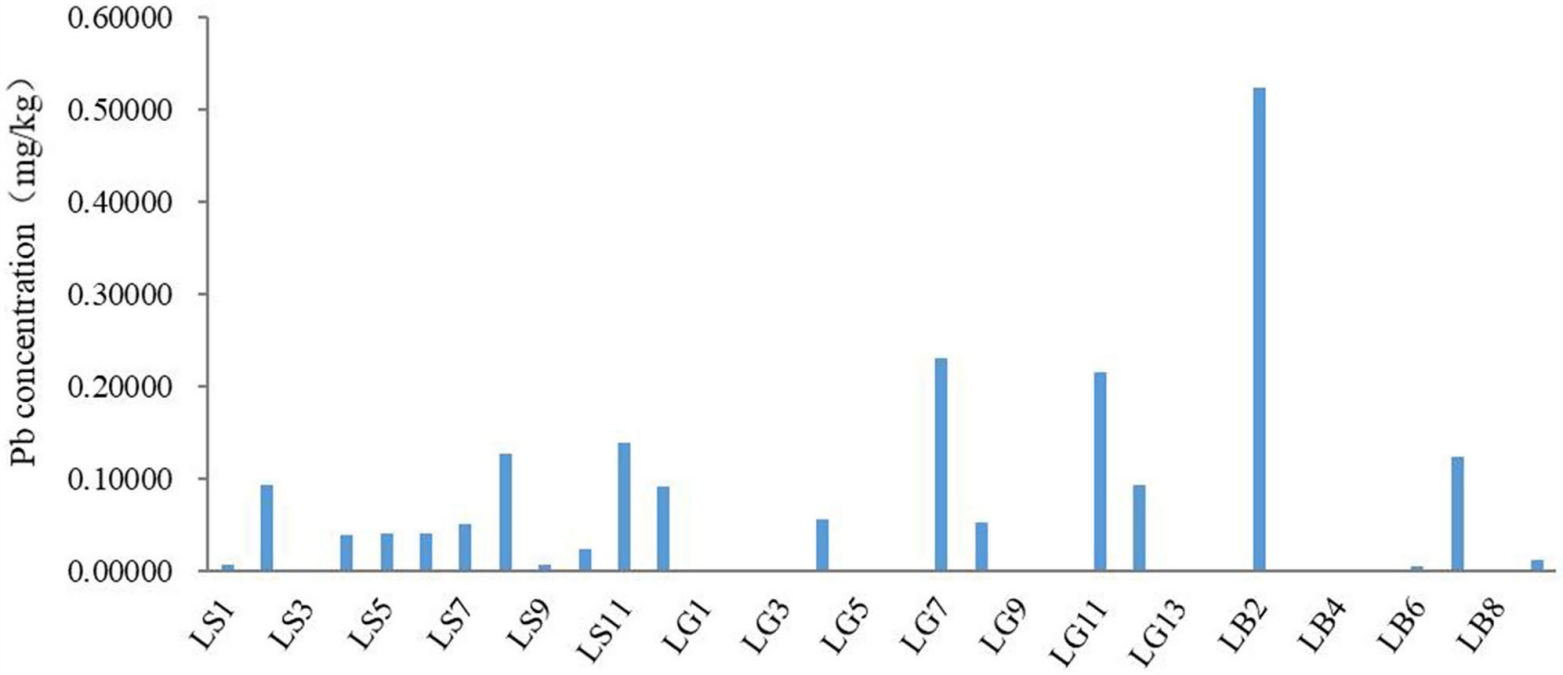
The contents of Pb in typical popular lip cosmetics (*N* = 34).

The Pb content in 34 lip products was determined in this study, as shown in Figure 3; Table 3. It can be seen from Figure 3 that the Pb content varies greatly among different lip products. In this study, the Pb element was not detected in about 41.18% lip products. At present, different countries, including China, the United States and Canada, have set limits for Pb in cosmetics, which are 10 mg/kg. The contents of lip products in this study were far below the limit. Therefore, the trace metal Pb element in lip cosmetics is at an acceptable level.

**Table 3:** The contents of Pb in various lip products (mg/kg).

**Parameter**	**Lipstick (*n* = 12)**	**Lip glosses (*n* = 13)**	**Lip balms (*n* = 9)**	**Totals (*N* = 34)**
Average value	0.05482	0.04976	0.07380	0.05791
Standard deviation	0.04506	0.07922	0.16358	0.10146
Maximum value	0.13849	0.23093	0.52470	0.52370
Minimum value	0.00000	0.00000	0.00000	0.00000

According to Table 3, the content of Pb in lip products (*N* = 34) ranged from 0 to 0.5237 mg/kg, which was far lower than that reported in previous studies such as South Korea (34), the United States (78), Iran (65), Saudi Arabia (20) and Turkey (18). This may be related to the batch, color, raw materials, and ratio of lip cosmetics (36, 79, 80). The average Pb content of all kinds of lip products decreased in the following order: lip balms (*n* = 12) > lipsticks (*n* = 13) > lip glosses (*n* = 9), while the result of Iwegbue et al. showed that the content of Pb in lipsticks is more than in lip glosses/lip balms (17). Probably, it reflected differences in the origin, color, brand and batch of lip cosmetics. In addition, in order to investigate the relationship between the content of Pb, the category and the price of lip products, this study carried out correlation analysis in SPSS. The results showed that the correlation coefficient between Pb content and category was 0.067 (*P* = 0.707 > 0.05), and the correlation coefficient between Pb content and price was −0.136 (*P* = 0.443 > 0.05). Therefore, there is no significant correlation among the content of Pb, the category and the price of lip products, which corroborate the findings of little relevance between the cost of cosmetics and the concentration of heavy metals (81). However, these results differ from some published studies which indicated that the concentration of Pb in cheaper brands was higher than the expensive brands (82, 83). This discrepancy could be attributed to the differences in limited sample selection.

### Carcinogenic and Non-carcinogenic Health Risk Assessment

**Figure 4:**
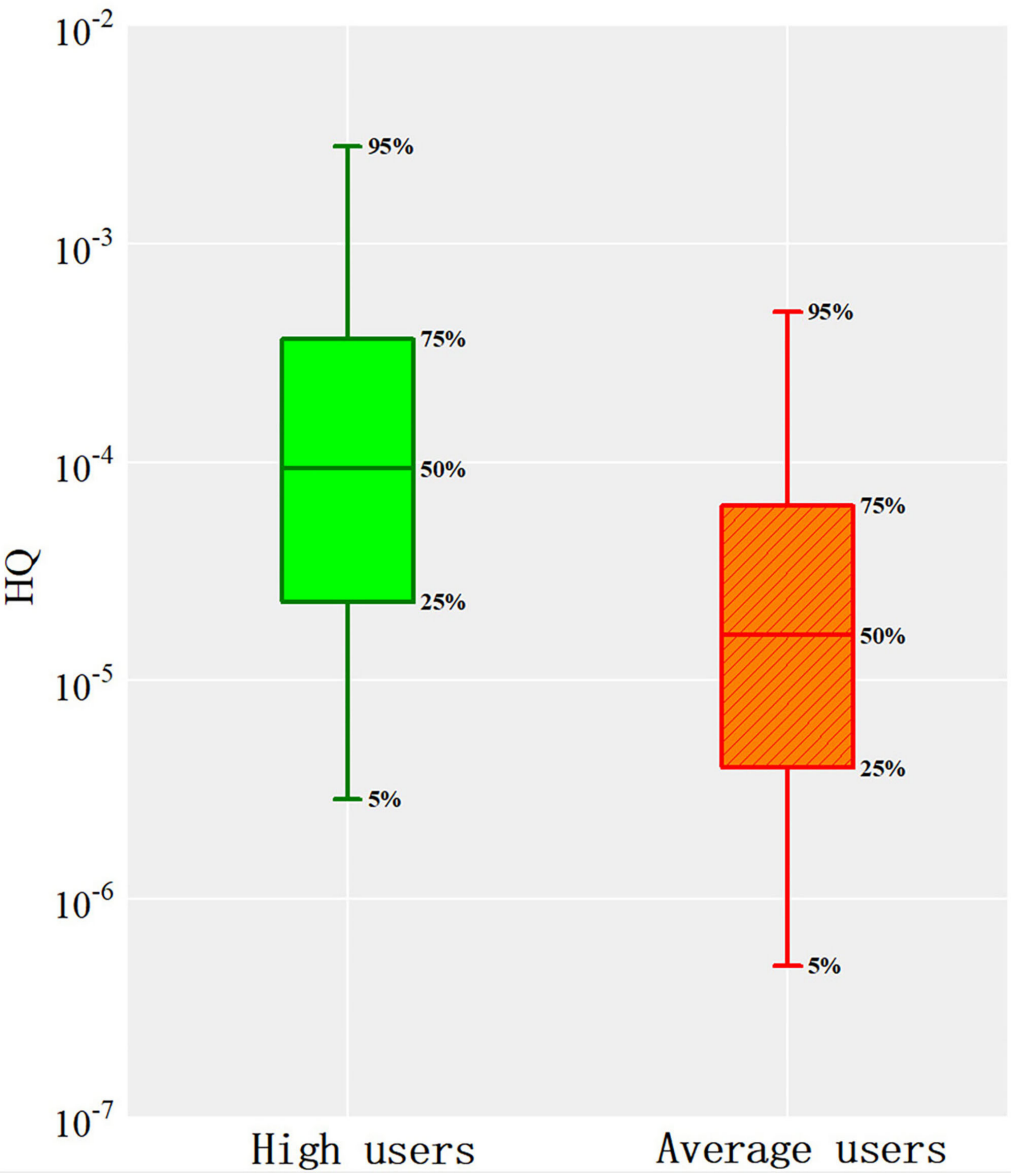
The non-cancer health risk assessment of Pb.

**Figure 5:**
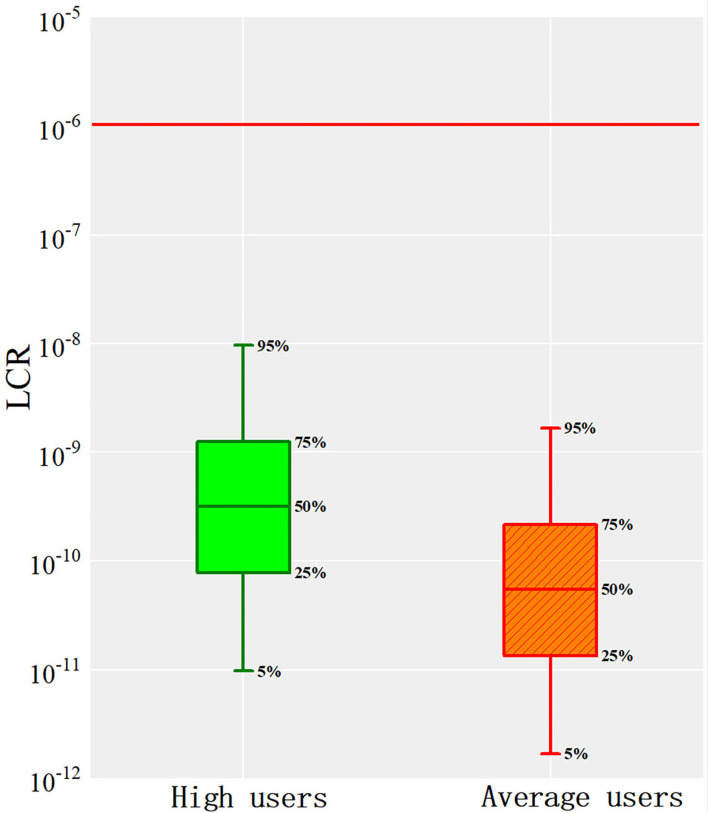
The cancer health risk assessment of Pb.

To evaluate the uncertainty caused by the difference in sample content, Monte Carlo algorithm was introduced for analysis. The results showed that the Pb content in lip cosmetics in this study was in line with the lognormal distribution. Combined with the health risk assessment model, 10,000 iterations were carried out to predict the final non-carcinogenic and carcinogenic risks, as shown in Figures 4, 5.

The non-carcinogenic risk prediction results of Pb in lip cosmetics showed that the 95th percentiles of high users and average users were 2.82 × 10^−3^ and 4.88 × 10^−4^, respectively, which were far below the threshold 1. Thus, the non-carcinogenic risk of exposure to Pb in lip products is acceptable, this result is consistent with previous research conclusions (33, 34, 84).

From Figure 5, it appeared that the carcinogenic risk of Pb in lip cosmetics was between 10^−12^ and 10^−8^. The 95th percentiles of high users and average users were 9.59 × 10^−9^ and 1.66 × 10^−9^, respectively, which were far lower than the threshold of 10^−6^. Therefore, it can be considered that the carcinogenic risk of Pb in contact with lip products is at a safe level. Though the carcinogenic risk of Pb in contact with lip products and other cosmetics, in many previous studies, is lower than the threshold of 10^−6^ (34), there will be a requirement for attention if the risk is above 10^−4^. In this study, the exposure of Pb may be considered safe currently. However, with daily using of the cosmetics, the heavy metals may poison and accumulate in human body. Therefore, the data provided in this study may be important to the managerial organization of cosmetic safety.

In this study, the exposure and health risks of Pb in lip products are the concerned objects. Exposure of hazardous substances in lip products is the result of interaction of various heavy metals and pollutants (22, 78), and this comprehensive exposure may have higher health risks, which need to be further enriched and deepened in the following studies.

### Blood Lead Level Analysis of Blood Lead

The use of lip cosmetics may affect the blood Pb content in children, especially through maternal transmission. Hence, based on the calculation results of different data parameters of the ALM model, the influence of adult exposure on blood Pb concentration in infants was obtained, as shown in Table 4.

**Table 4:** The running results for adult blood Pb model.

**Exposure scenarios**	**Blood Pb levels BLL(μg/dl)**							
Background	1.62				2.03			
Lip cosmetic	0.0006		0.0035		0.0006		0.0035	
PB_L_	1.6206		1.6235		2.0306		2.0335	
From mother to fetus	1.3775	1.4585	1.3799	1.4611	1.7260	1.8275	1.7284	1.8301

The left half of the table shows the corresponding adult blood Pb concentration of every part with low background value, which the reference value 1.62 μg/dl. And the other half of that displays the blood Pb content affected by high background value, which is 2.03 μg/dl with the combination of soil and reference exposure. And line labeled “Lip cosmetic” presents the blood Pb content affected by lip products exposure, which is 0.0006 and 0.0035 for average and high users, respectively. Subsequently, the adults blood Pb concentration could be summarized, as shown in the next line of the table. Finally, based on the fact that the transmission rate, in general, can be set as 0.85 or 0.9, the blood Pb concentrations from the mother to the fetus can be obtained. The results showed that under the condition of low background value and the transmission rate was 0.85, the blood Pb concentrations from the mother to the fetus of the average user and the high user were 1.3775 and 1.3799 μg/dl, respectively. While the transmission rate under the children's blood Pb pharmacokinetic model was 0.9, the blood Pb concentrations from the mother to the fetus of the average user and the high user were 1.4585 and 1.4611 μg/dl, respectively. Similarly, under the condition of high background value, the blood Pb concentrations from maternal to fetal were 1.7260 and 1.7284 μg/dl for both average and high users at adult blood Pb transmission rate, respectively. Under the condition of IEUBK for children, the blood Pb concentrations from maternal to fetal were 1.8275 and 1.8301 μg/dl for both average users and high users, respectively. No matter the concentration of Pb in lip cosmetics under average or high exposure, there is little difference in blood Pb content from mother to fetus. Hence, the main factor affecting fetal blood Pb concentration is the maternal background environment.

When running the IEUBK program, the maternal body was set to 0, 1.41925, and 1.77800 μg/dl, respectively. Under the background of average exposure, 0.00190 μg/d Pb was ingested from lip cosmetics and 0.07819 μg/d Pb was ingested from lip cosmetics under the condition of high exposure. The changes of blood Pb concentration in 0~7 years old children under the above six conditions were found, as shown in Table 5. Even if the total intake of Pb content changed slightly, the operation results of blood Pb concentration under the six conditions were the same.

**Table 5:** The running results for the IEUBK model.

**Age (years)**	**Total Pb absorption (μg/d)**	**Blood Pb levels (μg/dl)**
0.5~1	9.694 ± 0.002	5.2
1~2	13.224 ± 0.002	5.9
2~3	13.574 ± 0.002	5.1
3~4	18.684 ± 0.001	6.0
4~5	16.679 ± 0.002	5.7
5~6	16.294 ± 0.002	5.1
6~7	16.048 ± 0.002	4.6

In developed countries, the BLL of children <6 μg/dl are considered to be relatively safe (85), and the international threshold for children's blood Pb is 10 μg/dl (86). There would exist embryonic development toxicity and the pregnant women are prone to miscarriage when the concentration of blood Pb reach the standard. With the rise of BLL, people gradually experience the impact of heme metabolism, which can lead to renal dysfunction and even death (48, 87). Based on the data obtained in this study, although the blood Pb concentration in children did not exceed the international threshold, the blood Pb content of children aged 3~4 years old had reached the standard value in developed countries. In addition, there was little difference between the blood Pb data from the mother and the lipstick separately and the total of them. The results indicated that whether the average-exposed or the high-exposed population, the background Pb exposure is the main factor affecting the BLL of children. The Pb exposure of lip cosmetics did not significantly increase the blood Pb content of children, this conclusion is consistent with previous studies (48, 88). Compared with previous study, Monnot et al. found that the baseline BLLs of child born to mothers with high BLLs were 0.04 μg/dl higher than the BLLs of children born to mothers with average BLLs, and the lipstick exposure has little contribution to resulting in a higher level of BLLs than the dose of background exposure (48). Hence, more attention should be paid to the Pb content of air and soil around the living environment of children, as well as the safety of food and drinking water for adults and children.

### Uncertainty Analysis

A large number of studies have shown that heavy metal content in different lip products varies greatly, which may be related to origin, brand, color, price, raw materials and ratio (55, 63). Therefore, there is great uncertainty in sample selection. The selection of samples plays a fundamental role in the investigation of lip products, to effectively reduce the uncertainty of sample selection, Python crawler technology, is innovatively introduced in this study to select the best-selling brands in typical e-commerce platforms, which is in accord with the reality of market and consumers, making sample selection more objective and representative (89–91). The data of the e-commerce platform are updated in real-time observation. In order to carry out long-term effective risk control, it is necessary to continuously monitor the pollution levels of Pb and other heavy metals in lip cosmetics.

Furthermore, the risk levels appeared to be affected by metal contents. In the process of implementing risk assessment, this study introduces Monte Carlo simulation and predicts the risk assessment results through 10,000 iterations, which can reduce the uncertainty of risk assessment to some extent. Further investigations can obtain reliable data through short-term observation experiments on population exposure parameters, and more accurately measure the health risk of lip cosmetics.

Finally, it should be noted that this study assumes that the smeared lipstick is human intake, the absorption rate is 100%. In fact, due to the limitation of human body absorption, it is difficult to reach 100%. There are few existing studies regarding human body absorption, which brings certain uncertainty to risk assessment. Further work on human body absorption rate is particularly important.

## Conclusions

This study intends to investigate the heavy metal Pb content, assess health risks and analysis blood Pb level in popular lip cosmetics across Chinese e-commerce market. Python crawler technology, particularly, was introduced to identify and select the most popular 34 lip cosmetics. The findings clearly found that the average content of Pb in the tested lip products is 0.05791 mg/kg, far below the limit value. And the contents of Pb in various lip products decreased in the following order: lip balms > lipstick > lip glosses. There is no significant non-carcinogenic and carcinogenic risk caused by adult exposure to lip cosmetics. Furthermore, blood Pb model analysis showed that the exposure of Pb in lip cosmetics had little effect on children's blood Pb health. Compared with the Pb content of lip cosmetics, it is necessary to pay more attention to the safety of children's diet, drinking water and living environment to prevent the heavy metals from causing health damage.

## Data Availability Statement

The raw data supporting the conclusions of this article will be made available by the authors, without undue reservation.

## Author Contributions

YL organized study, prepared datasets, performed the statistical analysis, and drafted the manuscript. CL designed the study, organized study, prepared datasets, and revision of the manuscript. YF contributed to organize study, prepare datasets, and draft the manuscript. ZL contributed to study design and revision of the manuscript. YZ contributed to prepare datasets, perform the statistical analysis, and draft the manuscript. KL, LJ, and YY organized study and revision of the manuscript. BY and YS contributed to datasets prepare and revision of the manuscript. All authors read and approved the final manuscript.

## Funding

This study was financially supported by National Social Science Foundation of China (19FJYY02), China Postdoctoral Science Foundation (2020M672420), and Special Fund for the Reform of Education and Teaching in Central Universities, Zhongnan University of Economics and Law (YZKC202143).

## Conflict of Interest

The authors declare that the research was conducted in the absence of any commercial or financial relationships that could be construed as a potential conflict of interest.

## Publisher's Note

All claims expressed in this article are solely those of the authors and do not necessarily represent those of their affiliated organizations, or those of the publisher, the editors and the reviewers. Any product that may be evaluated in this article, or claim that may be made by its manufacturer, is not guaranteed or endorsed by the publisher.
